# A simulation model of colorectal cancer surveillance and recurrence

**DOI:** 10.1186/1472-6947-14-29

**Published:** 2014-04-08

**Authors:** Johnie Rose, Knut Magne Augestad, Chung Yin Kong, Neal J Meropol, Michael W Kattan, Qingqing Hong, Xuebei An, Gregory S Cooper

**Affiliations:** 1 Case Western Reserve University School of Medicine, 11000 Cedar Ave., Ste. 402, 44106-7136 Cleveland, OH, USA; 2 Case Comprehensive Cancer Center, Cleveland, OH, USA; 3 Norwegian National Centre of Telemedicine and Integrated Care, University Hospital North Norway, Tromsø, Norway; 4 Massachusetts General Hospital, Boston, MA, USA; 5 Seidman Cancer Center, University Hospitals Case Medical Center, Cleveland, OH, USA; 6 Cleveland Clinic, Cleveland, OH, USA

**Keywords:** Colorectal cancer, Recurrence, Surveillance, Follow-up, Model

## Abstract

**Background:**

Approximately one-third of those treated curatively for colorectal cancer (CRC) will experience recurrence. No evidence-based consensus exists on how best to follow patients after initial treatment to detect asymptomatic recurrence. Here, a new approach for simulating surveillance and recurrence among CRC survivors is outlined, and development and calibration of a simple model applying this approach is described. The model’s ability to predict outcomes for a group of patients under a specified surveillance strategy is validated.

**Methods:**

We developed an individual-based simulation model consisting of two interacting submodels: a continuous-time disease-progression submodel overlain by a discrete-time Markov submodel of surveillance and re-treatment. In the former, some patients develops recurrent disease which probabilistically progresses from detectability to unresectability, and which may produce early symptoms leading to detection independent of surveillance testing. In the latter submodel, patients undergo user-specified surveillance testing regimens. Parameters describing disease progression were preliminarily estimated through calibration to match five-year disease-free survival, overall survival at years 1–5, and proportion of recurring patients undergoing curative salvage surgery from one arm of a published randomized trial. The calibrated model was validated by examining its ability to predict these same outcomes for patients in a different arm of the same trial undergoing less aggressive surveillance.

**Results:**

Calibrated parameter values were consistent with generally observed recurrence patterns. Sensitivity analysis suggested probability of curative salvage surgery was most influenced by sensitivity of carcinoembryonic antigen assay and of clinical interview/examination (i.e. scheduled provider visits). In validation, the model accurately predicted overall survival (59% predicted, 58% observed) and five-year disease-free survival (55% predicted, 53% observed), but was less accurate in predicting curative salvage surgery (10% predicted; 6% observed).

**Conclusions:**

Initial validation suggests the feasibility of this approach to modeling alternative surveillance regimens among CRC survivors. Further calibration to individual-level patient data could yield a model useful for predicting outcomes of specific surveillance strategies for risk-based subgroups or for individuals. This approach could be applied toward developing novel, tailored strategies for further clinical study. It has the potential to produce insights which will promote more effective surveillance—leading to higher cure rates for recurrent CRC.

## Background

Roughly two-thirds of the more than 140,000 Americans diagnosed with colorectal cancer (CRC) each year will be treated surgically with curative intent [1]. Approximately one-third of these will experience recurrence of the original disease or will develop a new primary (metachronous) CRC [2,3]. Median survival for those experiencing recurrence of original disease is around two years [4]. Ultimately, nearly 50,000 patients in the U.S. die each year from CRC [1].

Most patients treated curatively are placed on some type of surveillance program involving periodic follow-up testing to detect preclinical recurrence. For patients who will experience recurrence, prognosis, though generally poor, may be improved if detection occurs prior to symptom onset, particularly if surgical resection of metastatic disease is possible [5]. Reviews of the relatively small number of trials comparing two or more specific follow-up strategies have suggested that more intensive strategies tend to increase survival at five years, detecting recurrence about six months earlier than less intensive strategies [6-8] at a point where salvage surgery with curative intent is more likely to occur (10.7% vs. 5.7%; p = .0002) [7]. However, the strategies tested, the populations studied, and the study periods varied significantly between trials [6-8]. As such, drawing prescriptive conclusions regarding best practices on the basis of these data is difficult.

Meanwhile, in economic terms, CRC surveillance consumes significant resources. U.S. investigators found that five-year follow up can cost as much as US$16,942 per patient [9]. Another group in Europe reported surveillance costs of US$9,011 [10] to US$59,841 [11] per detection of a recurrence resulting in attempted curative salvage surgery.

Not surprisingly, consensus is lacking among expert panels on how best to follow these patients [12-18]. New surveillance trials are in progress, but results may be several years away [19-21]. When complete, these trials will provide valuable information but will have been able to examine only a small fraction of possible surveillance strategies.

Given the potential impact on quantity and quality of life and on health care costs, better tools are needed for informing decisions around postsurgical surveillance of colorectal cancer patients. By simulating the dynamics of recurrence in a population of patients, a realistic computer simulation model could function as a virtual laboratory within which an unlimited number of experiments comparing hypothetical surveillance strategies could be run *in silico* within a compressed timeframe.

Several models have incorporated dynamics of the adenoma-carcinoma sequence in order to compare the effectiveness of hypothetical *screening* strategies in patients without history of CRC [22-32]; these include three models used in the National Cancer Institute’s comparative modeling effort CISNET (Cancer Intervention and Surveillance Modeling Network) [33]. Fewer models though have simulated the events following diagnosis and treatment of CRC in order to compare postsurgical *surveillance* strategies [34-37]. None has captured the dynamics of recurrence in a way that accounts for disease progression during diagnostic delay and that considers the full range of possible metastatic sites.

Capturing the dynamics of CRC recurrence is a major methodological challenge mainly because of the difficulty of estimating parameters describing progression of recurring disease amid the censoring caused by medical and surgical interventions. In order to create a realistic model which allows assessment of any hypothetical surveillance strategy, one must be able to account for disease progression amid diagnostic delay. Here, we describe a new approach to modeling the interaction between natural history of CRC recurrence and early detection of recurrence through surveillance testing—an approach designed to allow the simulation of any potential surveillance strategy. We introduce a basic model we have developed which applies this approach, preliminarily estimate disease progression parameters by calibration based on published outcomes from a classic surveillance trial, and offer a quantitative validation of the model.

## Methods

### Overview of approach

The model itself is comprised of two interacting submodels: a continuous-time disease progression submodel and a discrete time Markov submodel of surveillance testing and re-treatment. In the disease progression submodel, the exact time to earliest recurrence detectability is pre-determined for each simulated patient who will recur based on random draws from an exponential probability distribution. A pair of formulas—both functions of the time to earliest detectability--determines the timing of the transition to unresectability and to the point of symptom onset. Once these pre-determinations are made, the discrete-time Markov surveillance and re-treatment submodel simulates scheduled visits for surveillance testing of asymptomatic patient as frequently as every three months. This submodel references the pre-determined timeline of disease progression to determine whether asymptomatic recurrences are detectable by testing during surveillance visits, and whether recurrences are considered potentially resectable versus unresectable at the time they are discovered. Recurrences may alternatively be detected in the interval between surveillance visits as a result of symptoms which prompt individuals to seek earlier care.

To simulate the impact of any combination and schedule of surveillance tests, the disease progression submodel must be capable—in the extreme—of simulating disease progression in the absence of surveillance. Since most data describing recurrence tends to be “contaminated” by the effect of testing to detect asymptomatic recurrence and by subsequent intervention, it is difficult to directly estimate certain key parameters underlying a disease progression submodel. Therefore, we use a calibration approach to estimate these parameters while using the surveillance and re-treatment submodel described above to control for the effect of a known surveillance regimen on observed disease progression. Once these disease progression parameters are estimated through calibration, the schedule of follow-up tests embodied in the surveillance and re-treatment submodel can be changed to simulate any number of hypothetical follow-up regimens. In the case of the current work, we use the surveillance regimen followed in the intervention arm of a published CRC follow-up trial to calibrate disease progression parameters. We then change the surveillance regimen to be consistent with the control arm of the same study and compare the predicted to the observed outcomes.

### Modeling disease progression

Each individual *i* is randomly assigned a future recurrence status (i.e. will recur at some point or not) and site based on random number draws from a uniform probability distribution with range 0 to 1. Anatomic sites of recurrence are described in terms of local tumor status (none, locoregional, anastomotic only, or intraluminal/metachronous) and metastasis status (none, liver, lung, or multiple/other organs). All recurrences are considered to “begin” at the time of initial treatment since, by definition, there would have been at least some small amount of cancerous tissue which remained despite treatment. Each individual who will recur is assigned a future time D_i_ which represents the earliest point in time at which the recurrence of patient *i* will be detectable through surveillance testing. D_i_ is assigned randomly to these individuals from a continuous exponential probability distribution with hazard rate r_d_, leading to a declining incidence of recurrence with increasing time from initial treatment at the population level [38]. The time U_i_ at which the case is no longer amenable to curative salvage surgery (point of *unresectability*) is defined as

$$U_{i}=D_{i}+{X_{\mathrm{du}}}^{*}{\left(1+r_{u}\right)}^{D_{i}}$$

Where x_du_ is the baseline (i.e. where D_i_ = 0) interval between D_i_ and U_i_ and r_u_ is the rate of change in this window. A positive value of X_du_ would represent the minimum possible width of the window between earliest detectability and unresectability given r_u_ > 0. Beyond the point of unresectability, the patient will only be eligible for palliation and will be assigned a life expectancy accordingly.

A non-trivial number of recurrences are discovered through work-ups precipitated by patient symptomatic presentations in the intervals between scheduled surveillance visits [39]. This proportion can be expected to grow in inverse proportion to the vigilance of follow-up regimens, and should thus be modeled. The point of recurrence symptom onset S_i_ for patient *i* is defined as

$$S_{i}=D_{i}+{X_{\mathrm{ds}}}^{*}{\left(1+r_{s}\right)}^{D_{i}}+\epsilon_{\mathrm{ds}}$$

**Figure 1:**
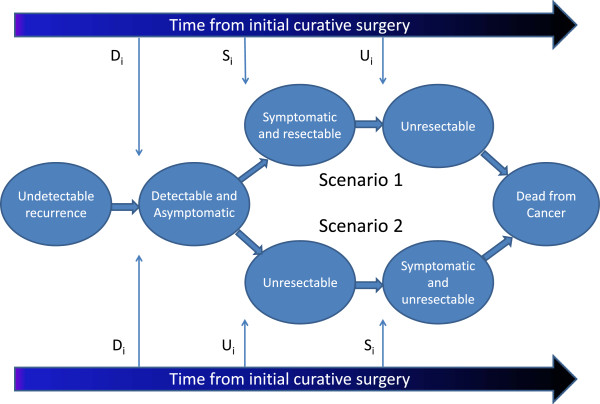
**Modeling disease progression in recurrent colorectal cancer.** Among the subset of individuals who will experience recurrence, cancerous tissue is considered to be present but undetectable until time D_i_. At time, S_i_, the patient will develop clinical symptoms of recurrence which will bring them to seek medical attention and will lead to diagnosis of recurrence if not previously detected. At time U_i_, the recurrent cancer will become “unresectable”: no longer amenable to curative treatment. U_i_ may be greater-than (Scenario 1) or less-than (Scenario 2) S_i_. In a scenario of no follow-up for detection of asymptomatic recurrence, the only patients for whom curative treatment of recurrence would be possible are those with S_i_ < U_i_ (Scenario 1) since symptoms would have brought them to seek medical attention at a point where curative re-treatment could occur. Continuous, heterogeneous values of D_i_, S_i_, and U_i_ are assigned to each patient who will recur based on the disease progression submodel as described in the text.

Where x_ds_ is the baseline (D_i_ = 0) interval between D_i_ and S_i_ and r_s_ is the rate of change in this window. A positive value of X_ds_ would represent the minimum width of the window between earliest detectability and symptom onset given r_s_ > 0. The normally distributed error term ϵ_ds_ (with mean = 0 and standard deviation = σ_ds_) is included to ensure sufficient variation in symptom onset so that some patients will develop symptoms before the point of unresectability and some will develop symptoms after that point. Figure 1 illustrates the two possible scenarios for progression of recurring individuals through clinically important points in disease natural history.

### Modeling surveillance and re-treatment

**Figure 2:**
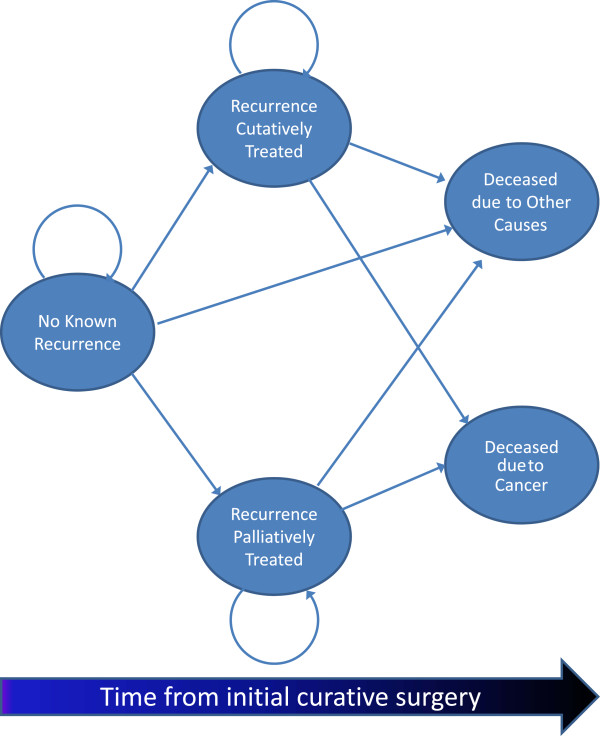
**Possible state transitions in the surveillance and re-treatment submodel.** An individual-based Markov submodel simulates the follow-up testing which may lead to detection of asymptomatic recurrence, and the treatment which may occur after recurrence diagnosis. The arrows represent possible state transitions which could occur at each three-month time step. Each patient begins the model in the “No known recurrence” state wherein they undergo surveillance testing according to a sepcified schedule. If a true recurrence is discovered, the patient will proceed to one of the two states: “Recurrence curatively treated” or “Recurrence palliatively treated”, depending on whether the current model cycle occurs before or after U_i_ (U_i_ is assigned as a continuous value based on the disease progression submodel). Patients in the “Recurrence curatively treated” state continue to undergo surveillance testing after treatment. During each cycle, individuals may move to the “Dead due to other causes” state from any other living state based on a background transition probability of mortality from other causes. Additional file 1: Figure S1 provides a more detailed depiction of the contingencies which drive state transitions in the surveillance and re-treatment submodel.

We used TreeAge Pro 2012 (Williamstown, MA) to create an individual-based Markov submodel of surveillance testing and re-treatment. Individual-based Markov models allow simulation of phenomena which involve transitions of heterogeneous individuals between states over time. During each discrete three-month time increment—or cycle—each of a series of hypothetical individuals may be in only one state at a time. Within a state, individuals may experience a series of probabilistic events which determine whether they will remain in their current state during the next cycle or transition to a new state. In our model, a series of simulated patients starts each cycle in one of five states: *no known recurrence, recurrence curatively treated, recurrence palliatively treated, deceased due to cancer, or deceased due to other causes* (See state transition diagram in Figure 2). Three months is used as a cycle length since this represents the shortest recommended inter-visit interval among published recommendations for post-surgical surveillance in CRC patients [12,15,16,40]. Note that only surveillance testing of asymptomatic patients occurs based on discrete time steps. Natural history events are measured in continuous time so that simulated patients will reach the point of detectability (D_i_), the point of unresectability (U_i_), and the point of symptom onset (S_i_) during the intervals between surveillance visits.

The time horizon of five years for surveillance was chosen because this is the timeframe over which virtually all recurrences manifest [7], and the majority of surveillance recommendations focus on this five-year horizon [12,15,16,40]. All individuals begin the first cycle—representing the period immediately following curative resection—in the *no known recurrence* state.

Each of the living states in the surveillance and re-treatment submodel is represented by a decision subtree (see Additional file 1: Figure S1). Whether or not a test is performed during a particular three-month cycle is dictated by a surveillance testing schedule matrix with a row for each three month cycle and a column for each possible testing modality. Using 1’s and 0’s, this matrix can specify any combination and timing of laboratory tests (e.g. carcinoembryonic antigen, or CEA), imaging studies (e.g. abdominal/pelvic CT), and provider visits. Positivity or negativity of each test given during a cycle depends on the current stage of the model compared to D_i_, the location of any detectable tumors, and the sensitivity and specificity of the test being employed. If any of the tests performed in a given cycle is positive, it is assumed that sufficient further workup using other available tests is undertaken to correctly identify detectable recurrence or otherwise rule out recurrence within that cycle with a combined sensitivity and specificity of 100%. Once recurrence is diagnosed (whether based on detection through scheduled surveillance or stemming from symptoms developing between scheduled visits), patients are assigned a life expectancy. The exact value depends on whether their recurrence has been diagnosed before or after reaching the point of unresectability (Table 1).

**Table 1:** Estimates for non-calibrated parameters

**Variable**	**Input parameter estimate**	**Range for sensitivity analysis**	**Source**^**a**^
**Screening test performance**			
***Carcinoembryonic Antigen test (CEA)***			
Sensitivity	.64	.49 - .79	[41]
Specificity	.90	.75 – 1.00	[41]
***Chest X ray***			
Sensitivity	.76	.61 - .91	[39,42,43]
Specificity	.95	.80 – 1.00	[43]
***CT – Hepatic metastases***			
Sensitivity	.83	.68 - .98	[44-47]
Specificity	.93	.78 – 1.00	[44,47]
***CT – Other abdominal metastases***			
Sensitivity	.46	.31 - .61	[44]
Specificity	.98	.83 – 1.00	[44]
***Hepatic ultrasound***			
Sensitivity	.62	.47 - .77	[47-49]
Specificity	.85	.70 – 1.00	[47-49]
***Colonoscopy***			
Sensitivity	.95	.80 – 1.00	[23,50]
Specificity	1.00	.85 – 1.00	[23,50]
***Clinical interview/examination***^**b**^			
Sensitivity	.42	.27 - .57	[5]
Specificity	.95	.70 – 1.00	[5]
**Life expectancy**			
After initial surgery, given no recurrence	20.7 years	--^c^	[51]
After recurrence with curative salvage	21 months	15 – 27	[52]^d^
After diagnosis of unresectable recurrence	8 months	4 – 12	[53]^e^

^a^When multiple sources are given, the parameter used in the model was estimated based on a sample-size-weighted mean.

^b^In the absence of laboratory or other diagnostic findings suggesting recurrence.

^c^Since the time horizon for the model was 5 years, varying this parameter over all but the most extreme low bounds would have no effect on results.

^d^Estimate based on incomplete reporting of survival among subjects undergoing curative salvage surgery in the Pietra study.

^e^Based on reported survival of 13 months following recurrence of colon cancer during the era of 1986–1992, and assumption that 1/3 of those relapsing will undergo curative salvage surgery with survival of 21 months as described above. The result is that those recurring but not undergoing curative salvage surgery would be expected to survive approximately 8 months after diagnosis of recurrence.

### Parameters

#### Calibration to estimate disease progression parameters

Values for the six disease progression parameters described above (r_d_, x_du_, r_u_, x_ds_, r_s_, and σ_ds_) are impossible or impractical to observe, and their values can be expected to differ based on disease and patient characteristics of the groups of interest. Therefore, we used a calibration process to estimate values for these parameters. We systematically ran the model with many thousands of different combinations of the unknown parameters in order to find the combination which yielded model outcomes most consistent with a real world data source [54]. As a data source, we used published data from a classic trial of an intensive versus a minimal follow-up strategy for patients who had undergone curative resection for CRC from 1987–1990 at an Italian Center [52]. Among all available trials of surveillance strategies, this study by Pietra et al. offered the most complete data needed for the exercise. Patients in the trial had been treated surgically for Astler-Coller Stage B or C CRC without adjuvant chemotherapy (additional details describing study participants in Table 2). The focus of the trial was on local recurrences; however, data for subjects with metastatic recurrences was also reported. We used data from the intensive follow-up arm to calibrate our model; accordingly, we refer to this group as the *calibration group*. To account for risk of non-cancer death among individuals who have not been diagnosed with recurrence, we calibrated an additional parameter (*m*) representing group-specific background (i.e. from non-CRC causes) mortality probability per cycle. We considered this a more reliable method to estimate background mortality in a small group compared to using life table-based estimates.

**Table 2:** **Comparison of calibration group and validation group based on intensive and minimal follow-up groups, respectively, from trial by Pietra et al.**[52]

**Value**	**Calibration group (Intensive follow-up)**	**Validation group (Minimal follow-up)**
**Group characteristics**		
Number of subjects	104	103
Male	56%	51%
Mean age at diagnosis	62.2 +/− 11	64.4 +/− 12
Astler-Coller stage B/C	59.6%/40.4%	58.3%/41.7%
Primary colon/rectal tumors	70.2%/29.8%	64.1%/35.9%
Preoperative complications	14%	11%
Recurrence rate during study period	39.4%	40.4%^a^
Distribution of metastatic disease if present: Liver/Lung/Other (includes multiple organs)^b^	26.7%/0.0%/73.3%	14.2%/4.8%/81.0%
**Surveillance testing schedule**		
Clinical interview/exam	Every 3 months for 2 years; every 6 months thereafter	Every 6 months for 1 year; every 12 months thereafter
CEA	Every 3 months for 2 years; every 6 months thereafter	Every 6 months for 1 year; every 12 months thereafter
Hepatic ultrasound	Every 3 months for 2 years; every 6 months thereafter	Every 6 months for 1 year; every 12 months thereafter
CT of abdomen/pelvis	Every 12 months	None
Chest x-ray	Every 12 months	Every 12 months
Colonoscopy	Every 12 months	Every 12 months
**Outcome targets**		
Disease-free survival at 5 years (DFS5)	68%	53%
Overall survival at one year after initial surgery (OS1)	97%	98%
OS2	90%	89%
OS3	84%	74%
OS4	76%	65%
OS5	73%	58%
Proportion undergoing curative salvage re-operation for recurrence	20%	6%

CEA = serum carcinoembryonic antigen assay; OSx = overall survival at x years.

^a^Includes a single metachronous (new primary) CRC

^b^Metastatic site was only reported by Pietra et al. for patients whose recurrences were metastatic only, and did not feature simultaneous involvement around the original tumor site.

We selected a set of seven observable outcomes reported by Pietra et al. to serve as *calibration targets* (note that these are distinct from the seven parameters estimated through calibration). The discrepancies between observed and model-predicted values of these targets were compared for each combination of values of the disease progression and background mortality parameters. The targets used were disease-free survival at five years (DFS5), overall survival at years one through five (OS1 – OS5), and the proportion of patients undergoing eventual curative salvage surgery following recurrence. Group characteristics and recurrence rates, surveillance testing schedule, and observed values for each of the calibration targets are shown for the intensive (calibration) group in the middle column of Table 2.

Searching the parameter space was achieved using a series of increasingly narrow grid searches. Specifically, each of the seven parameters to be calibrated was given an initially broad range (shown in the third column of Table 3) which was divided into three to eight evenly-spaced intervals. Values from across the initial range were systematically sampled so that all possible combinations of parameters were tried (i.e. a grid search of the parameter space [54]). Each run of the model simulated a cohort of 10,000 patients. Model--generated values of each calibration target were compared to values reported by Pietra et al. Candidate disease progression parameter sets which yielded target values > +/− one percentage point (or > +/− two percentage points if no values varying less than one percentage point were found) from the observed target value for DFS5 were eliminated from consideration. Next, model-generated and observed values for OS5 were compared in this same manner to further reduce the set of candidate parameter values, followed by OS4, OS3, proportion of patients undergoing curative salvage surgery, OS2, and finally OS1. OS2 and OS1 were considered last since these were most subject to influence from small numbers of events. The result was a small group of remaining parameter sets. Based on the range of candidate parameter values suggested by this process, a second round of calibration was performed, this time with a narrower set of candidate parameter values. This process was repeated for subsequent rounds until no additional gains in model fit—as judged by changes in the value of a goodness-of-fit statistic—were achieved. The goodness-of-fit statistic used for this purpose was a simple unweighted sum of squared differences between observed and predicted values for the calibration targets. The lowest goodness-of-fit value from among all sets of parameters from one round of calibration was compared to the lowest value from the previous round. Once no further improvement was seen between rounds, no further rounds of calibration were undertaken. After the final calibration round, the remaining parameter sets collectively contained a small range of candidate values for each parameter. A single best set was chosen which contained the values closest to the midpoint of the final range for each parameter (in the same order of priority which was applied throughout calibration: DFS5, OS5, OS4, OS3, probability of curative salvage operation, OS2, then OS1). This approach of using multiple, increasingly narrow grid searches was preferred on efficiency grounds to an approach of performing a single grid search with a large number of closely-spaced intervals for each parameter. With the latter approach, the number of parameters combinations would have been astronomical. Ultimately, we performed nine successive rounds of calibration, examining a total of 276,960 distinct parameter combinations (10,000 patients simulated for each parameter combination) in order to arrive at the final parameter estimates.

**Table 3:** **Starting ranges used in first round of model calibration – ranges were narrowed with successive rounds of calibration to achieve improved fit to the outcomes reported in the intensive follow-up arm of the Pietra trial**[52]

**Parameter**	**Definition**	**Starting range (inclusive)**	**Final calibrated parameter value**
**r**_**d**_	Rate per 3-month cycle at which recurrences transition from undetectable to detectable	0.05 - 0.12	0.092
**x**_**ds**_	Baseline (D_i_ = 0) presymptomatic window	1 week – 9 months	17 weeks
**x**_**du**_	Baseline (D_i_ = 0) window of resectability	1 week – 9 months	6 weeks
**r**_**s**_	Increment in presymptomatic window for each additional unit of D_i_ (expressed as a rate per 3-month cycle)	−0.025 - 0.15	0.07
**r**_**u**_	Increment in window of resectability for each additional unit of D_i_ (expressed as a rate per 3-month cycle)	0 - 0.15	0.11
**σ**_**ds**_	Standard deviation of normally-distributed error term for presymptomatic window	1 week – 6 months	11 weeks
***m***	Background five-year cumulative probability of mortality from non-CRC causes	1% - 20%	1.6%^a^

^a^By comparison, five year cumulative probability of death from any cause in the Italian population for a group with the age and gender makeup of the calibration group has been estimated at 6.1% for 1990 [51].

#### Non-calibrated parameters

Test sensitivity and specificity, as well as life expectancies, were estimated based on values from published studies (Table 1). Since evidence-based estimates were obtainable, calibration of these parameters was not necessary. Sensitivity and specificity values shown are for individual tests in isolation. It was assumed that metastases to multiple locations or to single sites besides the liver or lungs would be inoperable. It was also assumed that lung metastases were 50% as likely as liver metastases to be operable [55-57].

### Sensitivity analysis

One-way sensitivity analyses were performed on all non-calibrated model parameters according to the ranges shown in Table 1. The primary outcomes of interest in sensitivity analyses were OS5 and proportion of subjects undergoing curative salvage surgery for recurrence of CRC. The same randomization algorithm seed value was used throughout sensitivity analysis to minimize the impact of stochastic variability on comparisons with baseline model outputs.

### Validation

Once values for the six parameters describing the progression of CRC recurrence and the parameter representing background mortality for those without known recurrence had been estimated by calibration, the model was used to predict the outcomes that would be expected in the minimal follow-up arm of the Pietra trial; we will refer to this arm as the *validation group*. The primary purpose of this validation was to assess the model’s ability to predict cancer-related outcomes for a group with known disease characteristics and non-cancer mortality risk under a specified surveillance regimen. Keeping all calibrated disease progression parameters constant, the follow-up regimen modeled was modified to be consistent with that undergone by subjects in the validation group (see Table 2). Because the Pietra trial was not sufficiently large that subject characteristics were evenly distributed between groups through randomization, the proportion of patients relapsing and the distribution of metastatic sites in the model (see Table 2) were adjusted in validation to match what was reported for the minimal follow-up group. Specifically, probability of recurrence was adjusted from 39.4% to 40.4%, and distribution of metastatic disease between liver/lung/other was adjusted from 26.7%/0.0%/73.3% to 14.2%/4.8%/81.0%. Pietra et al. reported a substantial difference in DFS5 between the intensive and minimal surveillance groups (68% and 53% respectively). Only 1.0 percentage point of this difference was explained by a difference in recurrence rate. Since eventual cancer-related deaths do not affect disease-free survival calculation (rather, it is the *diagnosis* of recurrence which precedes death that counts), it was assumed that the remaining difference in DFS5 was attributable to differences in background mortality. We accordingly adjusted the five-year background cumulative probability of death from other causes in the validation group by 14.0%, equating to a 3.4 percentage point increment in annual mortality from other causes in the validation group compared to the calibration group. No adjustments were made to the structure of the model itself.

## Results

### Model calibration

**Figure 3:**
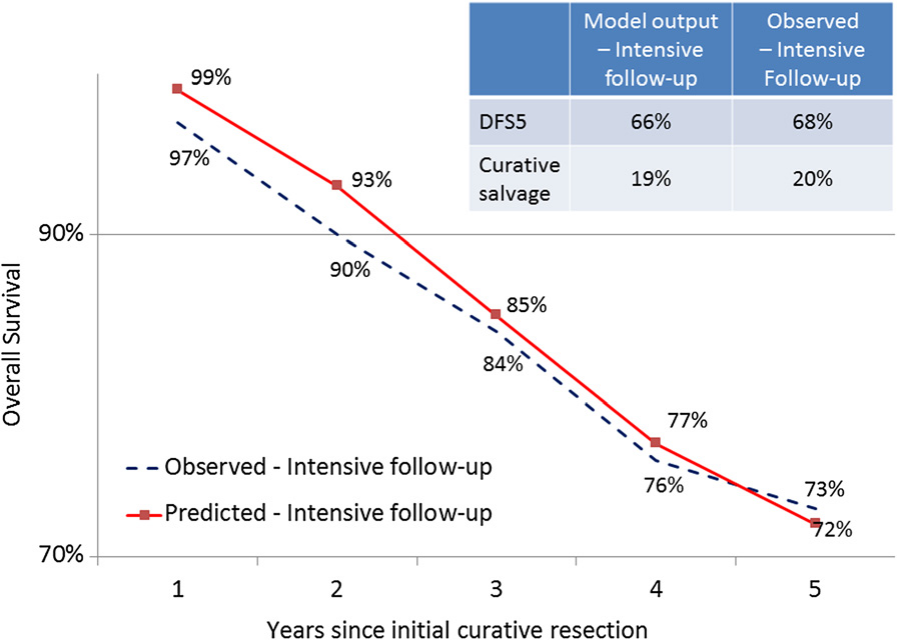
**Observed outcomes in intensive follow-up (calibration) group compared to outputs of fitted model.** DFS5 = disease-free survival at 5 years.

Final calibrated parameter estimates are shown in the fourth column of Table 3. Figure 3 compares observed values from the intensive follow-up (calibration) group in the Pietra trial to the model-generated outputs for the same group using the final best-fitting parameter set. As seen from the figure, predicted overall survival is slightly overestimated early in the five-year time horizon, while five-year disease-free survival and the rate of curative salvage surgery are slightly underestimated by the model. Additional file 2: Figure S2 depicts the disease progression milestones for 20 simulated patients with recurrence based on the calibrated parameters.

### Sensitivity analysis

In order to examine the sensitivity of key model results to changes in non-calibrated parameters, we conducted a deterministic sensitivity analysis, varying all non-calibrated parameters in the fitted model across the ranges shown in the third column of Table 1. The outcomes of interest for sensitivity analysis were the proportion of patients undergoing eventual salvage surgery (denominator = all patients initially treated for CRC, whether or not they eventually recurred) and OS5.

**Figure 4:**
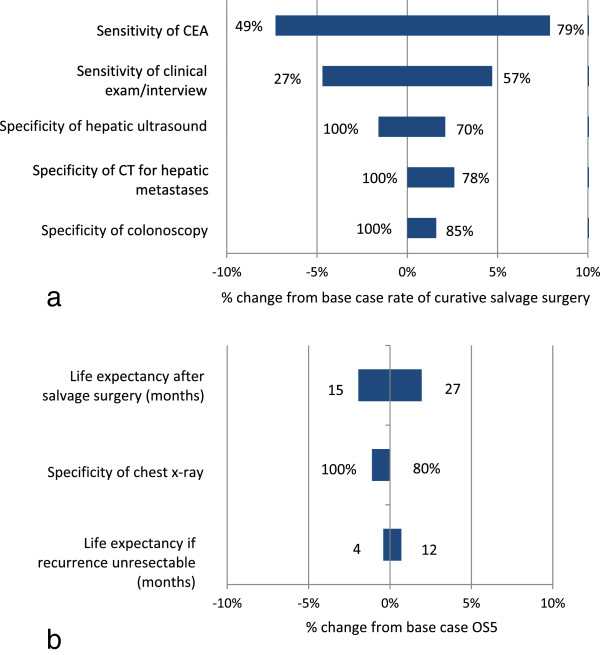
**One-way sensitivity analysis results. a)** When varied over the ranges indicated at either end of each horizontal bar, these parameters had the greatest impact on the predicted proportion of total patients undergoing curative salvage surgery for CRC recurrence within five years of initial treatment. **b)** Parameters with the greatest impact on predicted overall survival at five years (OS5).

Figure 4a depicts sensitivity analysis results for the outcome representing the proportion of patients undergoing curative salvage surgery. The most influential parameter was test sensitivity of the CEA assay, with a 15 percentage point increase in sensitivity to 79% resulting in a 7.9% increase in proportion undergoing curative salvage surgery, and a 15 percentage point decrease to 49% resulting in a 7.4% decrease in proportion undergoing curative salvage surgery. Varying all other parameters over the specified ranges yielded variation in curative salvage surgeries of within 5% of the base case.

Figure 4b shows sensitivity analysis results for the outcome of overall survival at 5 years. Life expectancy values had the greatest impact on this outcome, though no parameter proved highly influential.

### Validation

**Figure 5:**
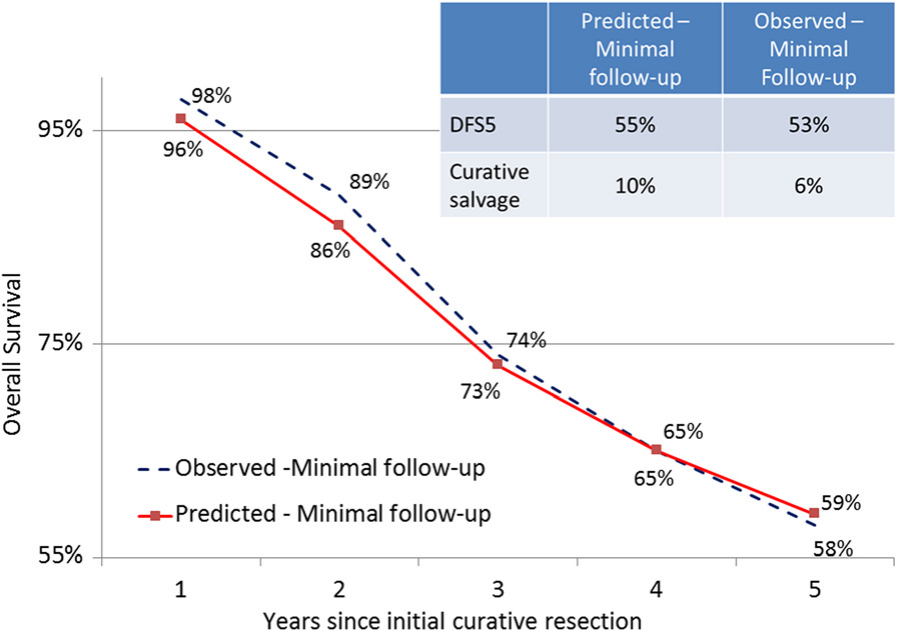
**Predicted vs. observed outcomes for the validation (minimal follow-up) group.** DFS5 = disease-free survival at 5 years.

In order to assess the predictive value of the model for a similar population under a different follow-up regimen, we ran the model for the minimal follow-up arm of the Pietra trial and compared model-predicted with observed outcomes. This comparison is shown in Figure 5. The largest difference between observed and predicted outcomes for the validation group was in the proportion of subjects who would eventually undergo curative salvage surgery for recurrence. The model overestimated this proportion at 10%, while only 6% of patients in reality underwent curative salvage surgery in the validation group.

## Discussion

We have introduced a new approach for simulating colorectal cancer surveillance and recurrence and have described a model which applies this approach. We estimated a best-fitting disease progression parameter set for this model by comparing iteratively generated model outputs to observed outcomes in one arm of a classic randomized controlled trial comparing two follow-up regimens (calibration). We then validated the model by demonstrating that it closely approximated several key outcomes for a separate group of patients followed less intensively in the same trial (validation). Application of this approach in subsequent based on larger, individual-level data sets could facilitate more personalized post-surgical surveillance of CRC survivors, ultimately translating into fewer low-yield tests and more lives saved through early detection of recurrence.

The approach of developing a generic disease progression submodel and calibrating its parameters based on outcomes data was taken because of the lack of biological data needed to directly estimate these parameters. The natural history behavior of recurring colorectal cancer is difficult to observe directly and is extremely heterogeneous. This heterogeneity likely stems, at least partly, from the greater genotypic diversity of genomically unstable tumor tissue which has been exposed to the stresses of treatment.

Though the specific values for the parameter estimates pertain to the patients in the Pietra trial and should not be construed to broadly represent disease behavior in the context of more recent treatment norms, they are consistent with generally-recognized patterns of CRC recurrence. Specifically, the estimates imply that those who recur later after initial treatment (higher values of D_i_) tend to exhibit a more indolent course of disease (as represented by a positive value of r_ds_) and are more likely to be considered curable when diagnosed (as represented by a positive value of r_du_) [53,58-60].

In sensitivity analysis, the proportion of patients undergoing curative salvage surgery was not highly sensitive to any single parameter. This is partly explained by the fact that approximately six of ten individuals did not recur; including these individuals in the denominator somewhat attenuates the variation in this outcome. However, even if only patients who recurred are included in the denominator, no parameter influenced the outcome by more than 20%. An interesting pattern did emerge. Probability of salvage surgery was most directly influenced by changes in sensitivity of tests which were not specific to an anatomic site (i.e. CEA and clinical exam/interview). In the simplest terms, these “tests” cast a wide net, with the potential to generate a true positive for any recurrence, regardless of site. This observation reinforces the rationale behind the current pursuit of newer, highly sensitive circulating markers for recurrence.

Paradoxically, proportion of curative salvage surgeries moved in inverse proportion to the specificity of those tests which targeted an anatomic site (e.g. hepatic ultrasound). This finding suggests that false positives for such tests actually provide an indirect benefit by precipitating further workups which diagnose true recurrences in other locations. This paradox underscores the importance of considering quality-of-life impact of testing as well as cost considerations. With these issues in mind, our model has been designed so that future versions may be used to compare the costs per quality-adjusted life year of alternative surveillance regimens. Specifically, the surveillance and re-treatment submodel has the ability to track costs due to surveillance testing, work-up of positive surveillance tests, and subsequent curative or palliative treatment for each simulated individual at each point in time. Quality-adjustment of life expectancy will be achieved by applying health state utility parameters to assigned life expectancies.

The fact that overall survival of CRC survivors in general is only modestly affected by changes in many of the parameters associated with diagnostic test characteristics and differential survival suggests an important implication. Because most survivors will not recur, and many of those who do recur have a narrow window within which early detection may change prognosis, developing tools to better identify those at greatest risk and to individualize their surveillance will likely yield greater benefit than applying generalized surveillance recommendations to all survivors. With calibration to larger, individual-based data sets covering patients with a range of risk factor profiles, it is hoped that future iterations of the model will provide a means to compare the effectiveness of proposed surveillance regimens for risk-based subgroups of patients and to even suggest optimized regimens for individuals.

Multiple models have been developed to evaluate CRC *screening* strategies in healthy populations. These models simulate the sequence by which benign polyps transform to adenocarcinomas, and by which these adenocarcinomas grow and invade healthy tissue [22-32]. Some of these screening models have spawned research questions involving disease natural history [28,61], have informed development of U.S. Preventive Services Task Force (USPSTF) guidelines on colorectal cancer screening [62], and have been applied by the Centers for Medicare and Medicaid Services (CMS) to compare the effectiveness of CRC screening strategies [63,64].

Only a few models have addressed the issue of *surveillance* of curatively treated CRC patients for early detection of recurrence. Two of these have not accounted for disease progression during diagnostic delay in a manner that would allow realistic assessment of novel surveillance strategies [34,35]. A separate group of investigators [36] used a sophisticated mechanistic, organ-level simulation to compare different treatment and surveillance strategies in patients with hepatic metastases. While the model does account for disease progression amid diagnostic delay, it does not provide information useful in determining optimal follow-up for non-hepatic recurrences. Also, the approach taken may be less feasible than a more population-based approach when considering metastases to other sites about which less detailed data are available.

A final, recent example of a model examining CRC surveillance is provided by Erenay et al. [37]. These investigators used longitudinal patient data to estimate natural history parameters related to formation and progression of metachronous colorectal tumors in patients who were status post curative resection. Though well-done, this model does not address recurrence of original cancers (which represent over 90% of relapses [7]). Furthermore, it is not validated against any other data source.

The approach we have described uses calibration of a disease progression submodel interacting with a surveillance and re-treatment submodel to estimate disease progression parameters while controlling for the impact of surveillance on disease natural history. The effect is to estimate group-specific parameters for a model describing disease progression in the absence of surveillance. Once calibrated, novel combinations and schedules of surveillance tests can be coupled with the disease progression model to predict outcomes for a group with similar underlying characteristics. Further calibration of the model to patient-level outcomes data containing individual risk factors will enable prediction of outcomes for populations or individuals with specific risk profiles, paving the way for more personalized and effective surveillance and the possibility for earlier detection of recurrence at a stage where cure is more likely.

### Limitations

The chief limitation of this work stems from the small size of the patient groups used in calibration and validation, and from the lack of individual-level data. With larger groups, the probability that underlying disease behavior and background mortality would be similar between populations used for calibration and populations for whom outcome predictions were being sought would be greater. We chose the Pietra trial because of the wealth of outcomes data it provided and the between-study-arm similarity in subject characteristics compared to other available published studies. In validation, we attempted to alleviate limitations related to group size by assigning the appropriate background mortality rates and distribution of metastatic sites to match those reported for the validation group. In a scenario of making actual population-level predictions, one might not have the luxury of knowing these characteristics of the group of interest. However, for our purposes, these adjustments can be seen as strengthening the internal validation of the model by controlling for known differences between groups. In the future, individual-level data describing more diverse groups will be used to provide more generalizable parameter estimates and a higher-resolution understanding of the effects of different risk factors. With larger sample sizes, far less variability can be assumed in metastasis pattern and background mortality between calibration populations and populations for which predictions are to be made.

A minor limitation relates to the fact that we did not explicitly model the occurrence of second primary (metachronous) CRC’s. Because no subjects in the calibration group were diagnosed with metachronous CRC, calibrating the model to simultaneously simulate incidence of metachronous CRC and recurrence of previously-treated disease was not a feasible option. Fortunately, the impact of this limitation on the integrity of the model validation should be minimal since only one individual in the validation group was reported to have experienced metachronous CRC. Because metachronous CRC (normally representing between 1.6% [7] and 7.4% [7,37,65] of CRC recurrences) behaves as a primary cancer and is associated with higher probability of cure compared to recurrent disease, it will be important to explicitly model separate natural history processes for these two types of events in future iterations of the model.

We do not consider the fact that the study is based on data gathered in the 1980’s and 1990’s to be a limitation. The purpose of this work was not to make prescriptive assertions about survivorship care of CRC patients today, but rather to demonstrate a method and preliminarily validate it using publicly-available data. That said, some useful generalizations are possible regarding the impact of diagnostic test characteristics and the importance of developing the ability to better customize follow-up of CRC survivors as elaborated above.

## Conclusion

Scheduled post-operative surveillance of patients curatively treated for colorectal cancer allows the detection of asymptomatic recurrence which can create the possibility of cure for some patients. We have described and preliminarily validated a simulation model, based on a novel approach, which allows comparison of important patient outcomes under hypothetical combinations and schedules of common surveillance tests.

### Future work

Next steps include adapting the model to a discrete event simulation framework which will allow calibration to individual-level patient data in which subject risk factors as well as precise dates of testing events and clinical milestones are known. Calibration and validation with larger, individual-level data sets representing patients with a more diverse range of demographic and disease characteristics is planned. Use of richer data sets (e.g. from combined clinical trials, or based on cancer registry data) will enable the use of future versions of the model to compare the effectiveness and cost-effectiveness of published surveillance recommendations for subgroups based on cancer stage, location (e.g. rectum versus colon), age, comorbidity status, presence of molecular markers, adjuvant treatment received, and other considerations. In addition, the model could be adapted to utilize an optimization algorithm to generate candidate follow-up strategies for such subgroups—allowing the more efficient design of meaningful clinical trials. Future adaptations could also power interactive clinical decision aids for planning the management of individual patients, an approach that itself could be validated in a randomized trial.

## Competing interests

The authors declare that they have no competing interests.

## Authors’ contributions

JR and KMA conceived the original idea for the work. JR developed the model with input from KMA, CYK, NJM, GSC, and MWK. JR, KMA, XA, and QH developed parameters estimates based on review of the literature. JR and CYK planned and performed model calibration and validation. JR wrote the manuscript with editorial input from all other authors. All authors read and approved the final manuscript.

## Authors’ information

JR is a Preventive Medicine physician and health services researcher in the Department of Family Medicine and Community Health at Case Western Reserve University School of Medicine. His main research focus involves simulation models of preventive interventions in cancer.

KMA is a practicing colorectal surgeon working at the University Hospital of North Norway. He serves as a research manager at the Norwegian National Centre for Integrative Care and Telemedicine, and was the principal investigator in a recently published randomized trail assessing colorectal cancer surveillance.

CYK is a researcher at the Massachusetts General Hospital Institute for Technology Assessment. He is an expert in methods for parameter estimation using calibration in complex models involving cancer.

NJM is the Dr. Lester E. Coleman, Jr. Chair Professor of Cancer Research and Therapeutics and Chief of the Division of Hematology and Oncology at University Hospitals Case Medical Center/Case Western Reserve University in Cleveland, OH. He serves as Associate Director of for Clinical Research at the Case Comprehensive Cancer Center, is Co-Chair of the National Cancer Institute Gastrointestinal Cancer Steering Committee, and serves on the Board of Directors of the American Society of Clinical Oncology (ASCO).

MWK is Chair of the Department of Quantitative Health Sciences at Cleveland Clinic in Cleveland, OH. His research is primarily focused on the development, validation, and use of prediction models in cancer.

QH is a graduate student in Biostatistics at Case Western Reserve University.

XA is a graduate student in Biostatistics at Case Western Reserve University.

GSC is a Professor of Medicine-Gastroenterology at University Hospitals Case Medical Center/Case Western Reserve University in Cleveland, OH. He directs the Office of Comparative Effectiveness Research at the Case Clinical and Translational Science Collaborative and co-leads the Cancer Prevention, Control and Population Research Program at Case Comprehensive Cancer Center.

## Pre-publication history

The pre-publication history for this paper can be accessed here:

http://www.biomedcentral.com/1472-6947/14/29/prepub

## Supplementary Material

Additional file 1: Figure S1 Simplified schematic of surveillance and re‒treatment submodel. For simplicity, only three testing modalities are shown in the figure: serum carcinoembryonic antigen (CEA) assay, CT of abdomen and pelvis, and chest x-ray. Other tests available in the model include chest CT, colonoscopy (for detection of second primary CRC’s), hepatic ultrasound, and clinical interview/exam. Life expectancies based on cancer-specific survival estimates (see Table 3) are assigned at the time of diagnosis/treatment. There is a probability of transitioning to the “Dead due to other causes” state during each cycle spent in any of the three living states. “Clones” are simply copied elements of the decision tree used to minimize tree size for display purposes (e.g. Clone 1: Full workup). Click here for file

Additional file 2: Figure S2. Scatterplot of disease progression for 20 simulated patients experiencing recurrence of previously‒treated colorectal cancer. These data points were generated using the calibrated parameter values for r_d_, x_du_, r_u_, x_ds_, r_s_, and σ_ds_ shown in the final column of Table 3. Individuals are ranked from earliest-recurring to latest-recurring. In this example, only patients #7 and #12 developed symptoms at a point where their recurrent disease would still have been curable. Note that connecting the green triangles would yield an approximate plot of the function Di, and that connecting the blue diamonds would yield an approximate plot if the function Ui. A fitted line through the red circles would approximate a plot of Si; there is significant deviation from such a line for individual red circles given the substantial calibrated value of σds, the standard deviation of the error term x used in calculating Si. In general, larger values of any of the rate (r) parameters would lead to more drastically curving lines, while lower values would yield straighter lines. Larger values of xdu and xds would lead to larger horizontal gaps between the lines representing Di and Ui, and Di and Si, respectively. Click here for file
