# Supplementary figures and images for: A simulation model of colorectal cancer surveillance and recurrence
Source: BMC Med Inform Decis Mak. 2014 Apr 8;14:29. doi: 10.1186/1472-6947-14-29 (PMC4021538; doi:10.1186/1472-6947-14-29)

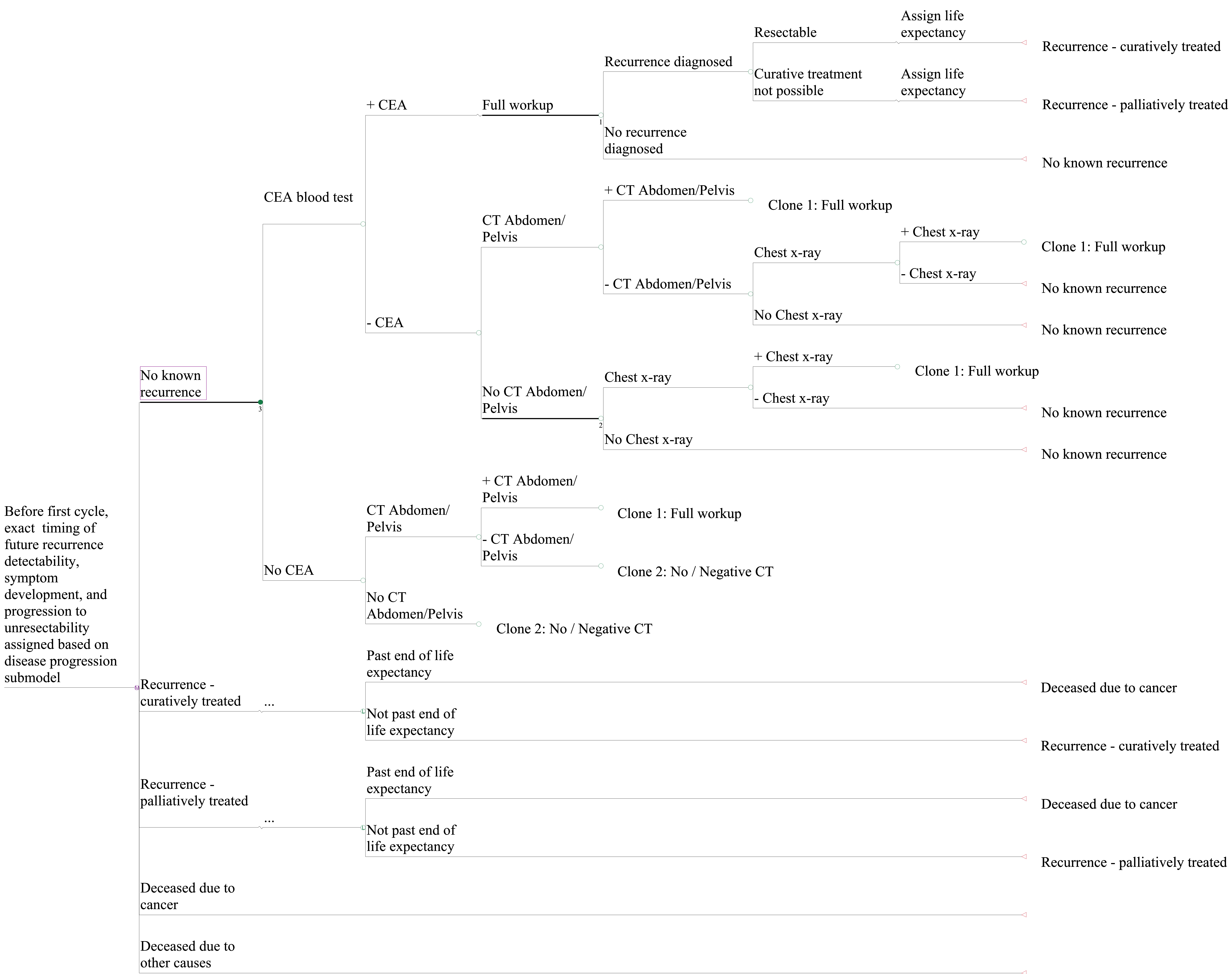

Supplement: Additional file 1: Figure S1 — Simplified schematic of surveillance and re‒treatment submodel. For simplicity, only three testing modalities are shown in the figure: serum carcinoembryonic antigen (CEA) assay, CT of abdomen and pelvis, and chest x-ray. Other tests available in the model include chest CT, colonoscopy (for detection of second primary CRC’s), hepatic ultrasound, and clinical interview/exam. Life expectancies based on cancer-specific survival estimates (see Table 3) are assigned at the time of diagnosis/treatment. There is a probability of transitioning to the “Dead due to other causes” state during each cycle spent in any of the three living states. “Clones” are simply copied elements of the decision tree used to minimize tree size for display purposes (e.g. Clone 1: Full workup). [file 1472-6947-14-29-S1.pdf]

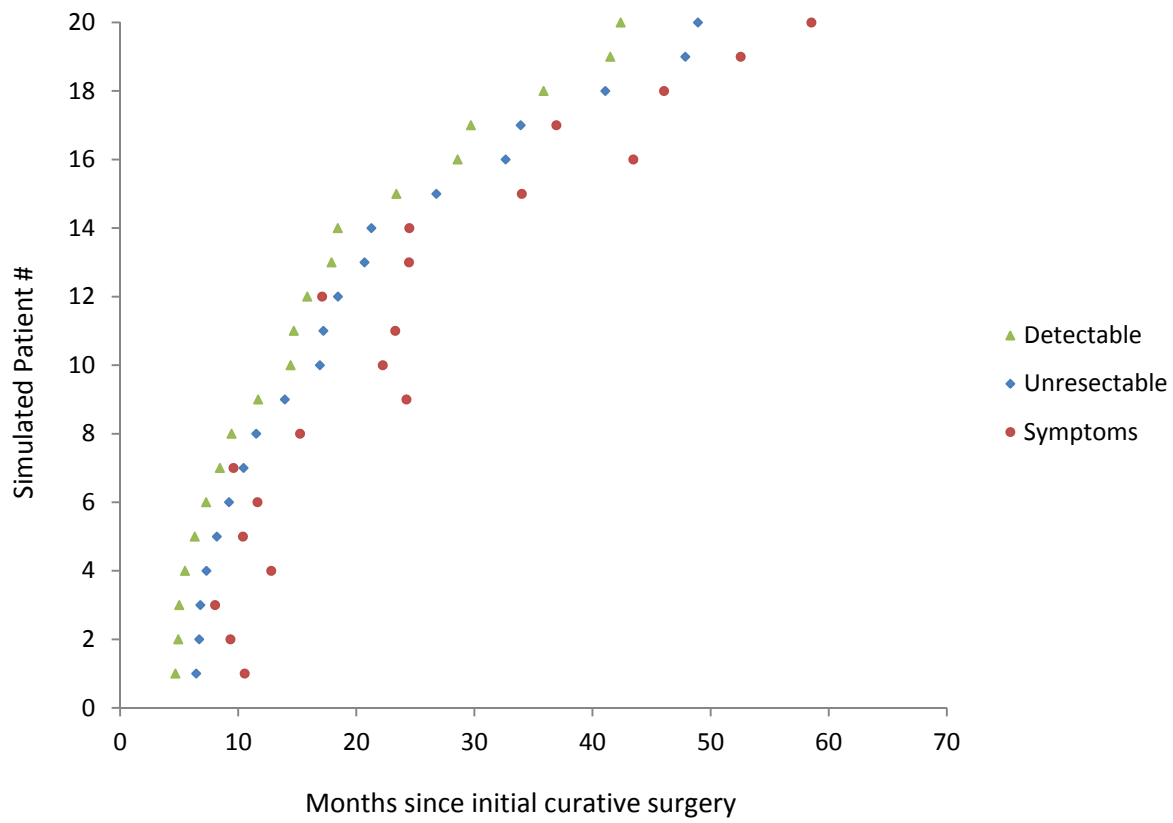

Supplement: Additional file 2: Figure S2. — Scatterplot of disease progression for 20 simulated patients experiencing recurrence of previously‒treated colorectal cancer. These data points were generated using the calibrated parameter values for rd, xdu, ru, xds, rs, and σds shown in the final column of Table 3. Individuals are ranked from earliest-recurring to latest-recurring. In this example, only patients #7 and #12 developed symptoms at a point where their recurrent disease would still have been curable. Note that connecting the green triangles would yield an approximate plot of the function Di, and that connecting the blue diamonds would yield an approximate plot if the function Ui. A fitted line through the red circles would approximate a plot of Si; there is significant deviation from such a line for individual red circles given the substantial calibrated value of σds, the standard deviation of the error term x used in calculating Si. In general, larger values of any of the rate (r) parameters would lead to more drastically curving lines, while lower values would yield straighter lines. Larger values of xdu and xds would lead to larger horizontal gaps between the lines representing Di and Ui, and Di and Si, respectively. [file 1472-6947-14-29-S2.pdf]
